# Supplementary material for: A Comparative Study of Chemical Profiling and Bioactivities between Thai and Foreign Hemp Seed Species (Cannabis sativa L.) Plus an In-Silico Investigation
Source: Foods. 2023 Dec 22;13(1):55. doi: 10.3390/foods13010055 (PMC10778124; doi:10.3390/foods13010055)
Supplement: Supplementary file 1 [file foods-13-00055-s001.zip › foods-2754374-supplementary.pdf]

Supplementary File

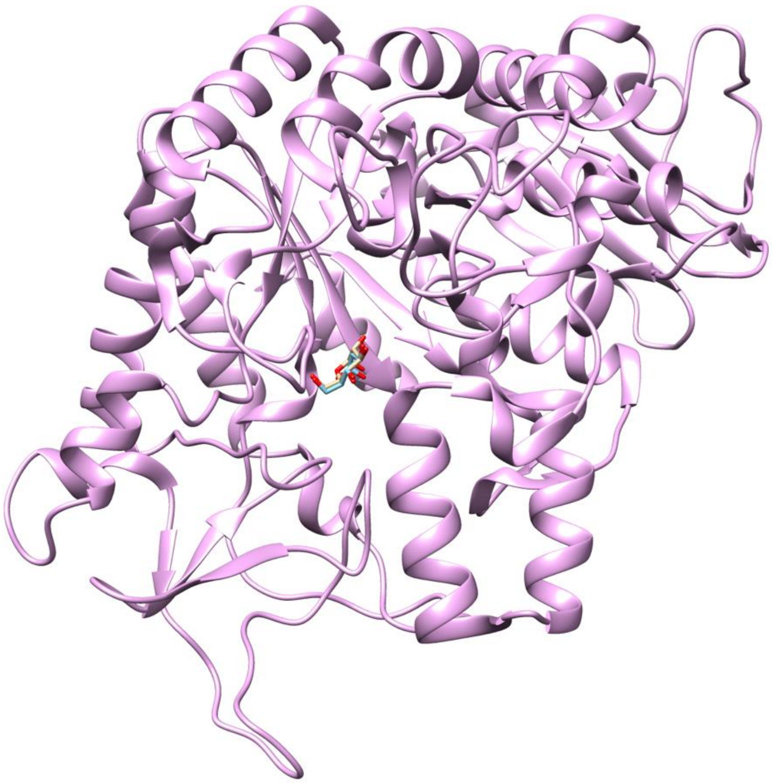

Figure S1. Docking validation of glucosidase enzyme (PDB ID 3A4A). The RMSD value of the re-docked (blue) and native (brown) glucose molecules is 2.715 Å (The accepted range is less than 3 Å).

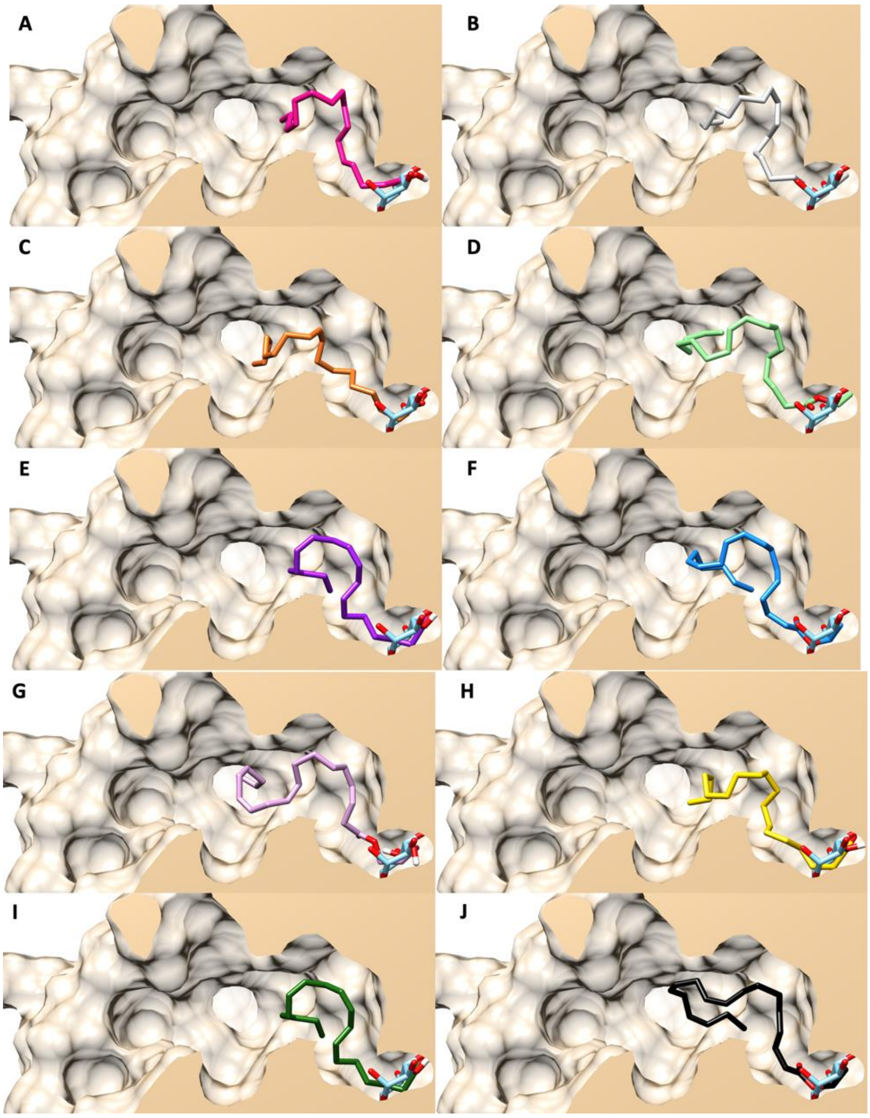

Figure S2. 3D docking poses of all fatty acids and their derivatives in close-up glucosidase catalytic pocket. A represents palmitic acid. B represents ethyl-palmitate. C represents oleic acid. D represents ethyl-oleate. E represents linoleic acid. F represents ethyl-linoleate. G represents glyceryl-linoleate. H represents steric acid. I represents linolenic acid. J represents ethyl-linolenate.

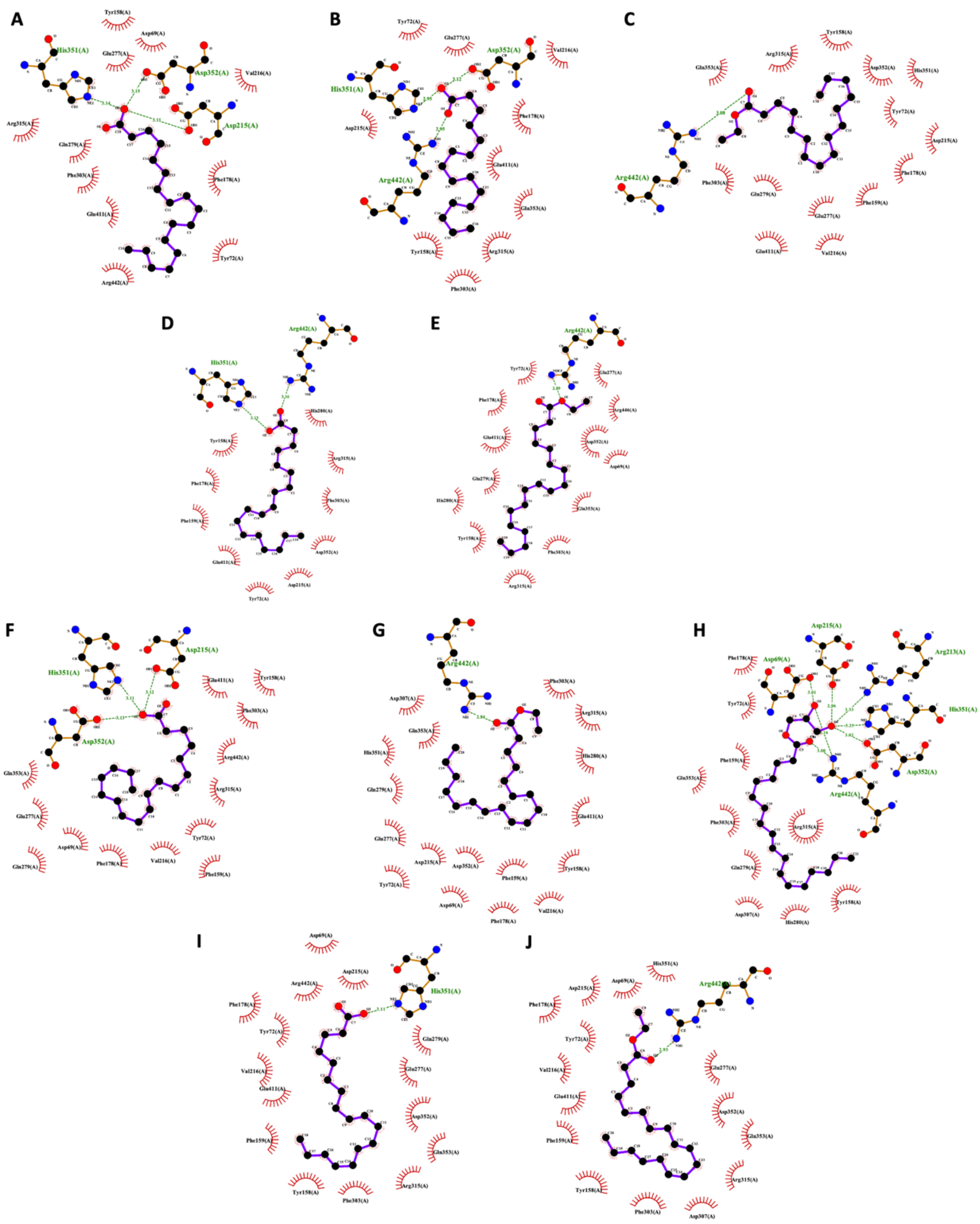

Figure S3. 2D interactions between docking poses of all fatty acids and amino acids in the glucosidase catalytic pocket. A represents steric acid. B represents Palmitic acid. C represents ethyl-palmitate. D and E represent oleic acid and ethyl-oleate. F and G represent linoleic acid and ethyl-linoleate. F, G, and H represent linoleic acid, ethyl-linoleate, and glyceryl-linoleate. I and J represent linolenic acid and ethyl-linolenate.

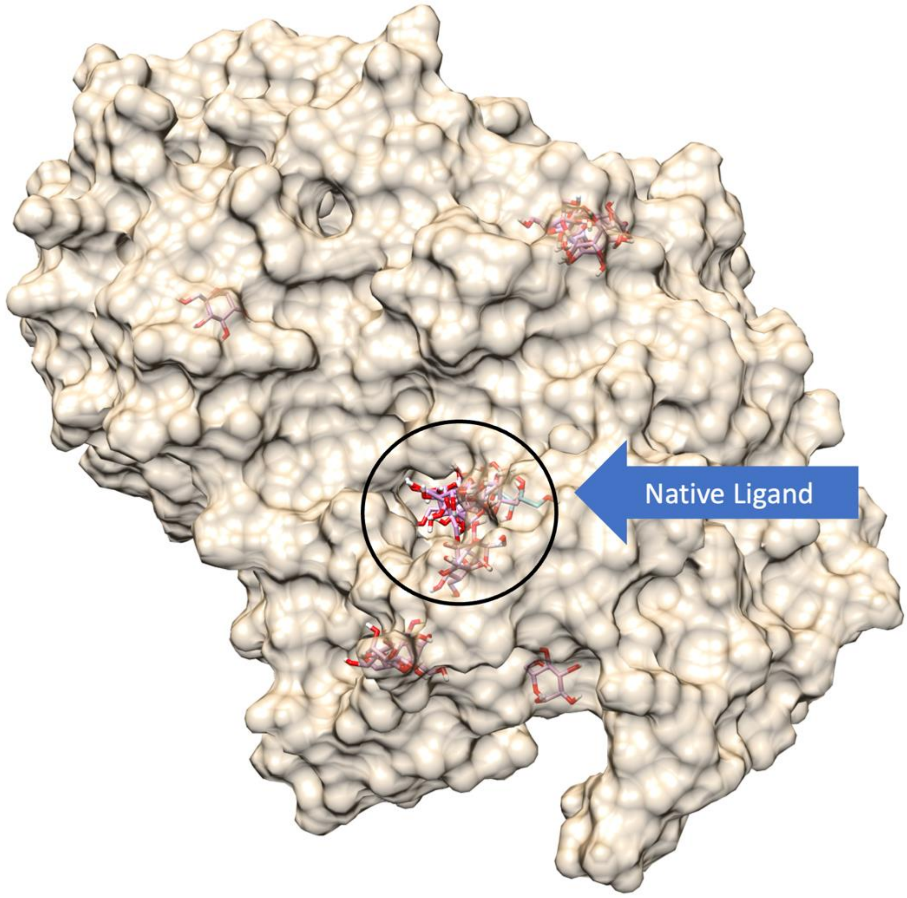

Figure S4. Additional docking validation for ligand-binding site prediction on glucosidase. Blue color ligand represents a native glucose molecule, while pink color ligands are docked glucose molecules. The black circle indicates a binding cluster with the highest docking poses, identical to the native ligand.

Table S1. Hemp seed samples and yield of different hemp parameter extractions.

No.	Sample	Weight (g)	Extract part	Weight (g) (Marc part)	Code	Weight (g)	% yield (w/w)
1.	RPF-1	2020.00	Oil	-	HS-TH-1-O	300.07	14.85
			Marc	651.39	HS-TH-1-M-H	58.43	8.97
				649.92	HS-TH-1-M-E	35.88	5.52
2.	RPF-3	2074.37	Oil	-	HS-TH-2-O	315.51	15.21
			Marc	848.52	HS-TH-2-M-H	63.28	7.46
				792.10	HS-TH-2-M-E	63.28	7.99
3.	FO#2	1958.16	Oil	-	HS-FS-1-O	298.77	15.26
			Marc	623.54	HS-FS-1-M-H	16.59	2.66
				546.06	HS-FS-1-M-E	33.50	6.13
4.	FO#3	900.00	Oil	-	HS-FS-2-O	172.48	19.16
			Marc	270.10	HS-FS-2-M-H	16.68	6.17
				271.90	HS-FS-2-M-E	16.80	6.17

Table S2. Antibacterial activity of hemp seed oils and extracts against the tested bacteria at a concentration of 2 mg/ml.

	HS-TH-1-O	HS-TH-1-M-E	HS-TH-1-M-H	HS-TH-2-O	HS-TH-2-M-E	HS-TH-2-M-H	HS-FS-1-O	HS-FS-1-M-E	HS-FS-1-M-H	HS-FS-2-O	HS-FS-2-M-E	HS-FS-2-M-H
SA	+	+	+	+	+	+	-	-	-	-	-	-
SE	-	+	-	+	+	-	-	+	-	-	+	-
MRSA	-	+	-	+	+	+	-	-	-	-	-	-
CA	-	+	-	+	+	+	-	+	-	-	+	-
EC	-	-	-	-	-	-	-	-	-	-	-	-
PA	-	-	-	-	-	-	-	-	-	-	-	-

SA: *Staphylococcus aureus* (ATCC 25923), SE: *Staphylococcus epidermidis* (TISTR 517), MRSA: Methicillin-resistant *Staphylococcus aureus*, (DMST 20654), EC: *Escherichia coli* (ATCC 25922), PA: *Pseudomonas aeruginosa* (ATCC 27853), CA: *Cutibacterium acnes*, HS: Hemp seed, TH: Thailand samples RPF-1 (HS-TH-1) and RPF-3 (HS-TH-2), FS: Foreign samples FO#2 (HS-FS-1) and FO#3 (HS-FS-2), O: Hemp seed oil; M: Marc, H: Hexane, E: 80% Ethanol

Table S3. Color list of glucosidase and ligands of interest used in the manuscript.

No	Name	Color	RGB System		
			R	G	B
1	Glucosidase	Tan	0.824	0.706	0.549
2	Native ligand	Light Blue	0.529	0.808	0.922
3	Palmitic Acid	Magenta	1.000	0.078	0.576
4	Palmitate, Ethyl ester	Grey	0.827	0.827	0.827
5	Steric Acid	Yellow	1.000	0.843	0.000
6	Oleic Acid	Organe	0.962	0.462	0.154
7	Oleate, Ethyl ester	Light green	0.565	0.933	0.565
8	Linoleic Acid	Purple	0.627	0.125	0.941
9	Linoleate, Ethyl ester	Dark Blue	0.118	0.565	1.00
10	Linoleate, Glyceryl ester	Pink	0.867	0.627	0.867
11	Linolenic Acid	Black	0.000	0.000	0.000
12	Linolenate, Ethyl ester	Dark Green	0.123	0.423	0.083

Table S4. Docking ligands - glucosidase's amino acid interactions list.

No	Compounds	Type	Residues
1	Palmitic acid (PA)	Hydrogen bond	HIS351, ASP352, ARG442
		Non-bond inteaction	TYR72, TYR158, PHE178, ASP215, VAL216, GLU277, PHE303, ARG315, HIS351, ASP352, GLN353, GLU411, ARG442
2	Palmitate, ethyl ester (PE)	Hydrogen bond	ARG442
		Non-bond inteaction	TYR72, TYR158, PHE159, PHE178, ASP215, VAL216, GLU277, GLN279, PHE303, ARG315, HIS351, ASP352, GLN353, GLU411, ARG442
3	Steric acid (SA)	Hydrogen bond	ASP215, HIS351, ASP352
		Non-bond inteaction	ASP69, TYR72, TYR158, PHE178, ASP215, VAL216, GLU277, GLN279, PHE303, ARG315, HIS351, ASP352, GLU411, ARG442
4	Oleic acid (OA)	Hydrogen bond	HIS351, ARG442
		Non-bond inteaction	TYR72, TYR158, PHE159, PHE178, ASP215, HIS280, PHE303, ARG315, HIS351, ASP352, GLU411, ARG442
5	Olecate, ethyl ester (OE)	Hydrogen bond	ARG442
		Non-bond inteaction	ASP69, TYR72, TYR158, PHE178, GLU277, GLN279, HIS280, PHE303, ARG315, ASP352, GLN353, GLU411, ARG442, ARG446
6	Linoleic acid (LA)	Hydrogen bond	HIS351
		Non-bond inteaction	ASP69, TYR72, TYR158, PHE159, PHE178, ASP215, VAL216, GLU277, GLN279, PHE303, ARG315, HIS351, ASP352, GLN353, GLU411, ARG442
7	Linoleate, ethyl ester (LE)	Hydrogen bond	ARG442

	Non-bond inteaction	ASP69, TYR72, TYR158, PHE178, ASP215, VAL216, GLU277, GLN279, PHE303, ARG315, HIS351, ASP352, GLU411, ARG442

No	Compounds	Type	Residues
8	Linoleic acid, glyceride (LG)	Hydrogen bond	ARG213, ASP215, HIS351, ASP 352, ARG442
		Non-bond inteaction	ASP69, TYR72, TYR158, PHE159, PHE178, ASP215, VAL216, GLU277, GLN279, PHE303, ARG315, HIS351, ASP352, GLN353, GLU411, ARG442
9	Linolenic acid (LNA)	Hydrogen bond	HIS351
		Non-bond inteaction	ASP69, TYR72, TYR158, PHE159, PHE178, ASP215, VAL216, GLU277, GLN279, PHE303, ARG315, HIS351, ASP352, GLN353, GLU411, ARG442
10	Linolenate, ethyl ester	Hydrogen bond	ARG442
		Non-bond inteaction	ASP69, TYR72, TYR158, PHE159, PHE178, ASP215, VAL216, GLU277, PHE303, ASP307, ARG315, HIS351, ASP352, GLN353, GLU411, ARG442
11	THC	Hydrogen bond	TYR158
		Non-bond inteaction	TYR158, PHE178, VAL216, GLU277, GLN279, PHE303, PHE314, ARG315, TYR316, ASP352, GLU411, ASN415, ARG442
12	THCV	Hydrogen bond	TYR158
		Non-bond inteaction	TYR158, PHE159, VAL216, GLU277, GLN279, PHE303, PHE314, ARG315, TYR316, ASP352, GLU411, ASN415
13	DTBP	Hydrogen bond	ARG442
		Non-bond inteaction	TYR158, PHE159, PHE303, ARG315, ASP352

Table S5. A detailed re-scoring docking function of fatty in this study.

		C16:00	C16:00e	C18:00	C18:01	C18:01e	C18:02	C18:02e	C18:03	C18:03e
No	Energy	PA	PE	SA	OA	OE	LA	LE	LNA	LNE
1	Final intermolecular Energy (kcal/mol)	-7.59	-7.81	-7.11	-6.43	-8.42	-6.65	-8.16	-6.65	-7.87
2	Final Total Intermal Energy (kcal/mol)	-1.53	-1.98	-1.74	-1.49	-2.02	-1.89	-2.34	-1.89	-1.89
3	Torsional Free Energy (kcal/mol)	+4.47	+4.77	+5.07	+4.47	+5.07	+4.18	+4.77	+4.18	+4.47
4	Unbound System's Energy (kcal/mol)	0.00	0.00	0.00	0.00	0.00	0.00	0.00	0.00	0.00
5	Estimate Free Energy of Binding (kcal/mol)	-4.65	-5.02	-3.77	-3.45	-5.37	-4.36	-5.73	-4.36	-5.29
6	EstimateKi (mM)	0.39	0.21	1.72	2.97	0.11	0.63	0.06	0.63	0.13

Table S6. A detailed correlation examination between previous experimental data and re-score docking energies.

No.	Name	Literature	1	2	3	4	5
		IC50 (uM)	Final Intermolecular Energy (kcal/mol)	Final Total Internal Energy (kcal/mol)	Torsional Free Energy (kcal/mol)	Unbound System's Energy (kcal/mol)	Estimate Free Energy of Binding (kcal/mol)
1	C16:0	13.97	-7.59	-1.53	4.47	0	-4.65
2	C18:0	11.34	-7.11	-1.74	5.07	0	-3.77
3	cis-C18:1	0.81	-6.43	-1.49	4.47	0	-3.45
4	C18:2	0.6	-6.65	-1.89	4.18	0	-4.36
5	C18:3	0.54	-6.65	-1.89	4.18	0	-4.36
Correlation R^2		1.0000	-0.9500	0.4117	0.6593	N.D.	-0.2465
